# Epigenetic mechanisms in breast cancer therapy and resistance

**DOI:** 10.1038/s41467-021-22024-3

**Published:** 2021-03-19

**Authors:** Liliana Garcia-Martinez, Yusheng Zhang, Yuichiro Nakata, Ho Lam Chan, Lluis Morey

**Affiliations:** 1 Sylvester Comprehensive Cancer Center, Miami, FL USA; 2 Department of Human Genetics, University of Miami Miller School of Medicine, Biomedical Research Building, Miami, FL USA

**Keywords:** Breast cancer, Endocrine system and metabolic diseases

## Abstract

The majority of breast cancers express the estrogen receptor (ERα) and agents targeting this pathway represent the main treatment modality. Endocrine therapy has proven successful in the treatment of hormone-responsive breast cancer since its early adoption in the 1940s as an ablative therapy. Unfortunately, therapeutic resistance arises, leading to disease recurrence and relapse. Recent studies increased our understanding in how changes to the chromatin landscape and deregulation of epigenetic factors orchestrate the resistant phenotype. Here, we will discuss how the epigenome is an integral determinant in hormone therapy response and why epigenetic factors are promising targets for overcoming clinical resistance.

## Abstract (web summary)

Endocrine therapy has been the mainstay for hormone responsive breast cancer treatment. Here, Garcia-Martinez and colleagues discuss epigenetic mechanisms regulating ER + breast cancer and endocrine therapy resistance, and highlight approaches to rewire the cancer epigenome to improve targeted therapies for this cancer.

## Introduction

Cancer is both a genetic and epigenetic disease. Epigenetic mechanisms regulate multiple aspects of cancer biology, from driving primary tumor growth and invasion to modulating the immune response within the tumor microenvironment. Unlike genetic mutations, which are challenging to correct, dysregulated epigenetic mechanisms can be feasibly targeted by small molecule compounds. Furthermore, modulation of the epigenome in various solid cancers exposes cancer cells to attacks by the immune system, increasing their sensitivity to immunotherapy^1,2^. These advantages generated a growing interest in the last decade to developing epigenetic strategies to combat cancer.

Epigenetics-based diagnostic and prognostic tools greatly contribute to precision oncology. Notably, several DNA methylation diagnostic screens are currently undergoing clinical trials or are already being used in the clinic^3^. Efforts in precision oncology to combat dysregulated epigenetic mechanisms also led to the development of epidrugs — drugs targeting epigenetic modulators. Currently, only nine epidrugs are FDA-approved, including inhibitors of EZH2, IDH, histone deacetylases (HDACis), and DNA methyltransferases (DNMTs) with many others undergoing clinical trials for treating solid (NCT01928576, NCT03179943) and hematologic tumors (NCT03164057, NCT02717884). Of note are the estrogen receptor-positive (ER^+^) breast cancer phase II trials (NCT04190056, NCT00828854, NCT00676663) testing efficacy of epidrugs in combination with traditional therapies, reflective of recent advancements in our understanding of the epigenetic mechanisms governing ER^+^ breast cancer growth, metastasis, and treatment resistance.

The incidence of invasive breast cancer has been increasing since 2004, with more than two million cases reported worldwide in 2018, and over 270,000 U.S. cases were projected for 2020^4^. Around 80% of all breast cancer cases are categorized as ER^+^ due to expression of ERα and these patients have a 5-year overall survival rate of around 90%^4,5^. Since ERα is the primary oncogenic driver in most ER^+^ cancers, current endocrine-based therapeutic options include ERα-blockade, estrogen synthesis inhibition, and selective ERα degradation. Although endocrine therapies extend overall survival, a third of all early-stage ER^+^ breast cancer patients will experience treatment resistance^6^. Targeting chromatin regulators with small compounds to rewire the cancer epigenome may re-sensitize resistant cells to endocrine therapy or induce sensitivity to novel treatments.

In this Perspective, we will introduce several key epigenetic mechanisms regulating the biology of ER^+^ breast cancer and discuss their contribution to therapeutic resistance. We will also highlight areas representing novel opportunities to improve targeted therapies for ER^+^ breast cancer. These approaches have the potential to revolutionize how we diagnose and prognose patients, devise personalized treatment strategies, and provide better care to patients with ER^+^ breast cancer.

### Estrogen subtypes and mechanisms of ERα signaling

Estrogen stimulates many developmental processes including reproductive maturation and bone growth as well as energy homeostasis in the body by modulating insulin sensitivity, the rate of feeding, and energy expenditure via thermoregulation. Estrogen also coordinates mitogenic and epigenetic mechanisms to regulate mammary gland development. There are five main estrogen subtypes: estrone (E1), 17-β estradiol (E2), estriol (E3), estetrol (E4), and estrone-sulfate (E1s). E1 is reversibly converted to E2, the more biologically active form, and both represent the main estrogens in the body. E3 and E4, however, are only detectable during pregnancy, with E3 predominating. E1s mainly serves as an estrogen reservoir, as it is easily converted in situ to its active forms, E1 and E2, via steroid sulfatases^7^.

**Fig. 1:**
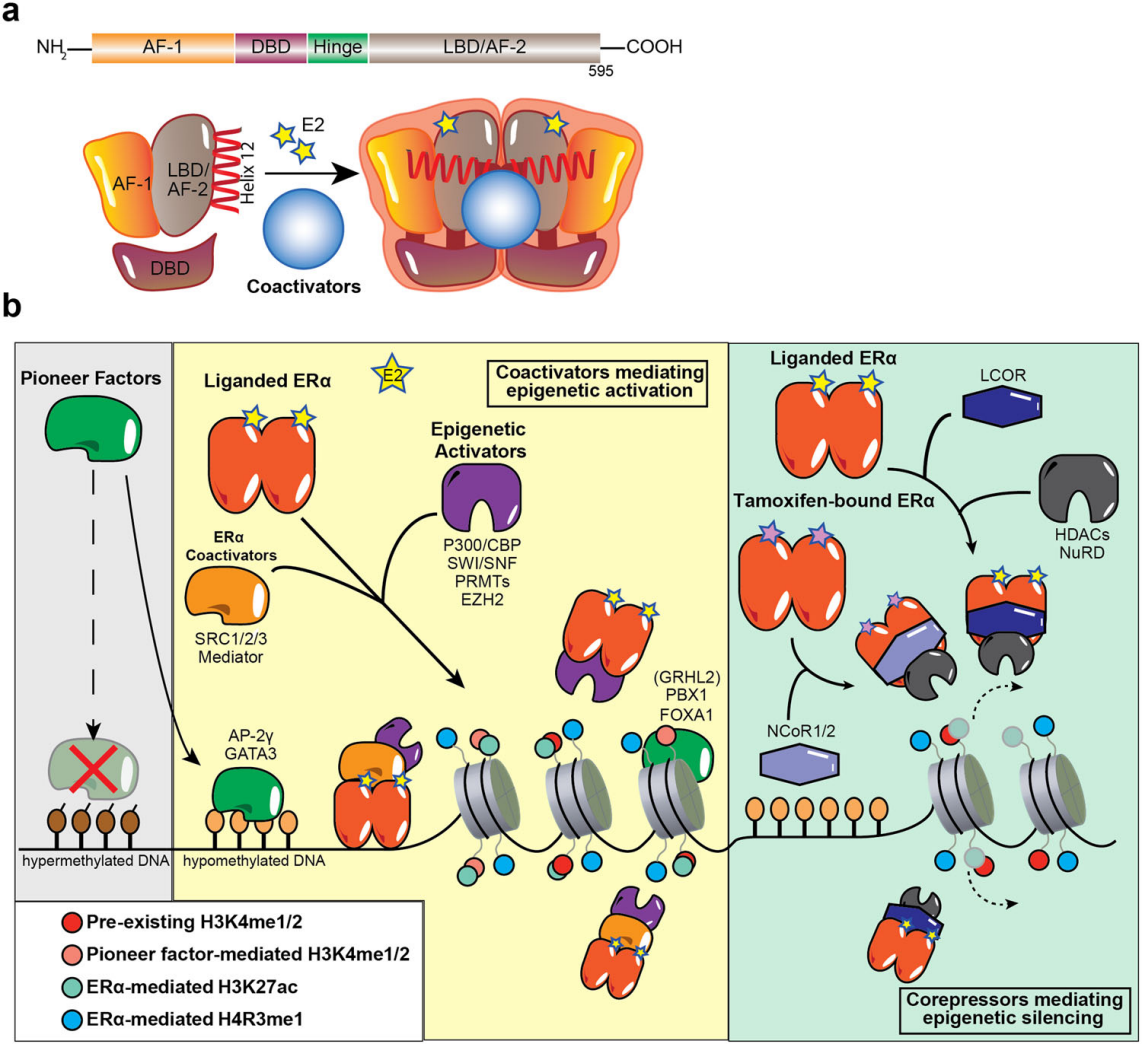
ERα mediates epigenetic changes by interacting and crosstalking with pioneer factors and co-regulators. **a** Schematic depiction of various structural domains within the estrogen receptor (ERα). The LBD harbors surfaces for dimerization as well as coactivator binding. Upon binding E2, helix 12 within the LBD shifts to an active conformation, which promotes ERα interaction with coactivators. **b** Pioneer factors such as FOXA1, GATA3, PBX1, and AP-2*γ* preferentially bind to hypomethylated genomic sites bearing their respective motifs. Importantly, these sites are often already marked with low levels of H3K4me1/2, which increase with the recruitment of pioneer factors such as FOXA1. Upon stimulation with the E2 ligand, pioneer factors facilitate the localization of liganded ERα to the chromatin. This leads to the activation of gene expression as ERα recruits epigenetic activators such as P300/CBP, the SWI/SNF complex, PRMTs and EZH2 (through direct contact or through coactivators such as SRC-1/2/3 and Mediator) to deposit activating epigenetic marks such as H4R3me1 and H3K27Ac (solid-colored). However, through interactions with corepressors such as LCOR and NCoR1/2, liganded ERα and tamoxifen-bound ERα can also recruit epigenetic repressors including HDACs and the NuRD complex to mediate gene repression by removing active epigenetic marks (faintly colored).

The structure of ERα is central to its ability to respond to E2 stimulation. It contains several functional domains that determine its transcriptional and epigenetic activities including the N-terminal activation function 1 (AF1), hinge domain, activation function 2 (AF2) within the C-terminal ligand-binding domain (LBD), and the DNA-binding domain (DBD)^8^ (Fig. 1a). The intrinsically disordered AF1 is a common phosphorylation target of mitogenic kinases to alter ERα transcriptional activity. ERα dimerization occurs on the LBD interface, which also binds E2, resulting in a conformational shift at helix 12 that activates the receptor. AF2, the major transcriptional activation domain, mediates co-regulator interactions based on helix 12 conformation. Between the N-terminal AF1 and C-terminal LBD is the hinge domain containing the nuclear localization signal to direct ERα to the nucleus. Finally, the DBD enables ERα to bind its consensus DNA sequence known as estrogen response elements (EREs).

ERα mediates E2-stimulated signaling either through genomic pathways that involve interactions with the chromatin or non-genomic pathways, which occur independently of ERα chromatin recruitment. Non-genomic E2 signaling pathways control up to 25% of ERα target genes and directly promote ER^+^ breast cancer cell proliferation^9^. Importantly, the most rapid effects of E2 stimulation occur within minutes and are due to non-genomic E2-mediated activity. Cytoplasmic signaling pathways such as MAPK and PI3K/AKT also regulate liganded-ERα signaling^10^. Indeed, these two well-characterized non-genomic mechanisms of crosstalk between ERα and mitogenic pathways promote therapeutic resistance in ER^+^ breast cancer, discussed in a later section.

Genomic-mediated mechanisms following E2 stimulation begin with ERα homodimerization and recruitment to chromatin either directly to EREs or indirectly by tethering to transcription factors (TFs) (e.g., SP1, FOS, and JUN; NF-kB; and C/EBPβ) via its AF domains^10^. It is estimated that up to 75% of estrogen-responsive genes require ERα binding to EREs or ERE-like sequences for their expression^11^. In ER^+^ breast cancer, pioneer TFs like FOXA1, GATA3, PBX1, and AP-2γ bind specific DNA target sequences in condensed chromatin and facilitate ERα chromatin binding in response to E2 stimulation. Activated ERα can also recruit a cohort of coactivators or corepressors to mediate gene transcription or repression (Fig. 1b).

### Epigenetic mechanisms underlying ERα signaling

Upon E2 stimulation, hundreds of ERα coregulators are recruited to the chromatin in a highly coordinated manner to ensure the proper transcriptional and repressive activity at ERα target sites. We and others found that ERα cycles on and off the chromatin in the order of minutes and hours, although each molecule of ERα dwells on the chromatin for only seconds at a time upon E2 stimulation^12–15^. Prominent epigenetic ERα coactivators comprise members of the p160 family, P300/CBP, SWI/SNF complex, PRMTs, and the Mediator complex (Fig. 1b). SRC-1, SRC-2, and SRC-3 of the p160 family of coactivators directly bind ERα and act as a platform for ERα to recruit other activating enzymes and chromatin remodeling complexes to modify the epigenetic landscape at targeted enhancers and promoters^16^. P300, a histone acetyltransferase (HAT), is recruited to ERα-bound enhancers via interactions with SRC proteins, namely SRC-3, to acetylate lysine 27 of histone H3 (H3K27ac), thereby activating the enhancer^17,18^.

Although H3K27ac signal at ERα-bound sites do not change on average upon acute E2 stimulation, we and others found that E2 increases H3K27ac levels at sites where ERα exhibits significant regulatory functions^12,13,15,19^. Coincident with increasing H3K27ac at ERα-bound enhancers is the recruitment of BRG1, the catalytic component of the SWI/SNF chromatin remodeling complex, suggesting that ERα recruits the SWI/SNF complex to further remodel and activate enhancers^20^. Notable corepressors of ERα transcriptional activity include NCoR1, NCoR2, and LCoR, that bring epigenetic repressors into contact with ERα to mediate downregulation of E2-repressed genes^21^ (Fig. 1b). BRCA1 is perhaps the most well-known ERα corepressor. Upon binding the AF2 domain, BRCA1 monoubiquitinates ERα, targeting it for degradation, thereby downregulating ERα transcriptional activity^22^. The epigenetic and oncogenic roles of these and other coregulators are reviewed extensively^23–25^.

Pioneer TFs are required for E2-dependent ERα recruitment to chromatin. They bind to chromatin independently of E2 and their depletion significantly reduces E2-induced ERα chromatin binding. The epigenetic and oncogenic roles of pioneer TFs in ER^+^ breast cancer are reviewed^26^. Although not yet considered a pioneer TF, we postulate that GRHL2 displays many functional similarities to FOXA1 and GATA3, such as E2-independent recruitment to chromatin and regulation of ERα target genes, suggesting its potential role as a pioneer TF^27–29^. Last, we and others recently revealed that PRC1 and PRC2 components exhibit E2-dependent chromatin recruitment and promotes E2-induced ERα target gene expression in breast cancer cells^15,30–33^. We reviewed the repressive and activating functions of Polycomb complexes in different cellular contexts and their mechanisms in stem cells, development, and cancer previously^34^. Additional in-depth studies of GRHL2 and Polycomb-group proteins in the context of ER^+^ breast cancer will be needed to fully characterize their roles as regulators of ERα signaling.

### Epigenetic processes in normal mammary gland development are derailed in breast cancer

Mammary gland development is mediated by a plethora of signaling pathways and chromatin regulators as well as hormonal clues that coordinate the balance between self-renewal, differentiation, and tissue integrity. The mammary gland develops through three major stages: embryonic, pubertal, and reproductive. Embryonic mammary gland development is coordinated by signaling pathways such as WNT and Hedgehog (HH), while the pubertal and reproductive stages are under hormonal control^35^.

**Fig. 2:**
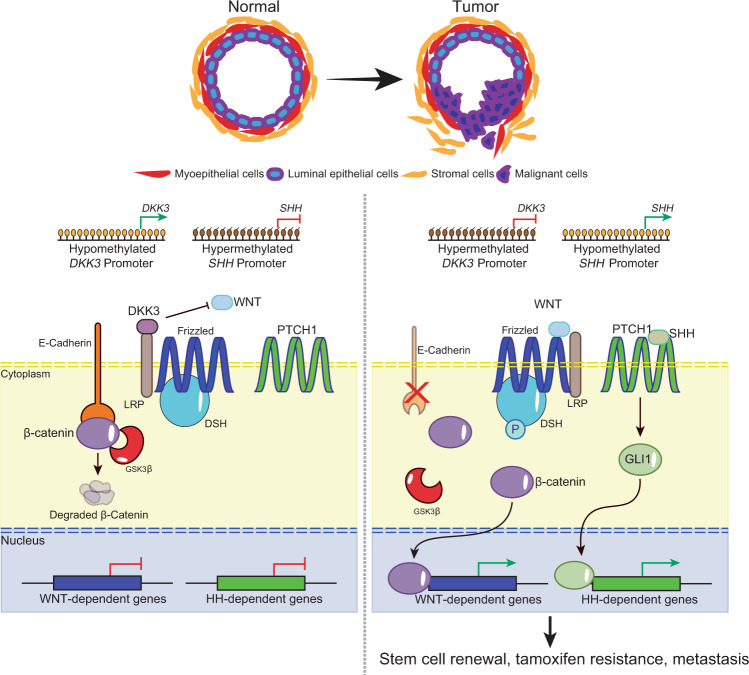
The development of embryonic mammary glands is dependent on carefully coordinated spatial-temporal activation of signaling pathways such as WNT and Hedgehog (SHH). In normal mammary epithelium, DKK3 binds to LRP, a WNT pathway coactivator of Frizzled, which prevents the activation of the pathway in the presence of the WNT ligand. E-Cadherin binds to cytoplasmic β-catenin, which is degraded by GSK3β in the absence of WNT activation. The promoter of *SHH* (encoding the Hedgehog ligand SHH) is hypermethylated and the Hedgehog pathway is silenced. In breast cancer, however, the *DKK3* promoter is hypermethylated, which leads to its downregulation. In the absence of DKK3, LRP can coactivate Frizzled in the presence of the WNT ligand, leading to phosphorylation of DSH, which inhibits GSK3β from degrading β-catenin. E-Cadherin is also downregulated via promoter methylation. In addition, the *SHH* promoter becomes hypomethylated, thereby upregulating the expression of SHH and activating the Hedgehog pathway via GLI1. Activation of the WNT and the Hedgehog pathways lead to stem cell renewal, EMT, metastasis, and tamoxifen resistance.

Reactivation of developmental pathways is a common feature in different types of cancer and, in breast cancer, is closely related to the maintenance of the mammary gland stem cell population^36^ (Fig. 2). Studies in the last decades, with the advent of technological advancements such as next-generation sequencing, revealed that derailment of epigenetic processes important during mammary development also plays a significant role in breast cancer progression. Here, we discuss the functional crosstalk between epigenetic processes and developmental signaling pathways that contribute to breast cancer.

### Epigenetic modulation of WNT signaling in ER^+^ breast cancer

Aberrant WNT signaling activation leads to the genesis and progression of several cancer types including breast. Epigenetic silencing of WNT antagonist genes, including *SFRP* and *DKK*, contributes to breast tumorigenesis^37^. Mechanistically, silencing of these genes through DNA methylation is a major cause of continuous WNT signaling in breast cancer and is associated with poor prognosis^38^. These alterations lead to constitutive activation of β-catenin resulting in increased stem cell renewal and proliferation that is associated with disease relapse^37^ (Fig. 2). Interestingly, in a study of 96 breast cancer samples, promoter methylation of *DKK3*, a member of the DKK family, was significantly enriched in tumors from patients with advanced stage disease, lymph node metastasis, and positive ERα status (42 of 47 samples were ER^+^)^39^. Given that the WNT and ERα signaling pathways are connected, notably via the Polycomb protein EZH2^31^, we speculate that the lack of WNT inhibition by DKK can feed forward into the ERα signaling pathway (and vice versa) to promote growth and survival, thereby correlating *DKK3* promoter methylation with positive ERα status. Interestingly, the use of agents such as 5-azacytidine and trichostatin A restores *DKK3* expression in vitro^39^. Efforts to restore ERα expression in the clinic with hypomethylating agents, though, have not been successful. However, targeting the derailed epigenetic regulatory circuit leading to activation of developmental programs such as WNT signaling should be explored. Concepts such as enhancer switching, a normal process occurring during development that regulates the switch in the transcriptional activation of key survival genes between cancer stem cells and differentiated cells^40^, suggest that we can modulate WNT signaling using epigenetic agents to mirror the regulatory mechanisms present in normal development.

### Crosstalk between epithelial–mesenchymal transition (EMT) and methylation of DNA and histones

E-cadherin (encoded by *CDH1*) regulates intracellular localization of β-catenin, and silencing of *CDH1* can result in aberrant WNT/β-catenin signaling and EMT^41^ (Fig. 2). EMT modulates mammary epithelial cell polarity, vectoral flow of milk during pregnancy, and cell movements during wound repair. It is a reversible process dynamically controlled by a framework of TFs including ZEB1, SNAIL, and TWIST as well as epigenetic machineries. For instance, SNAIL recruits the DNA methyltransferase DNMT1 to repress *CDH1* through DNA methylation. Furthermore, reactivation of *SNAIL* transcription by TGFβ-induced EMT is controlled by the H3K27me3 demethylase KDM6B. Notably, *SNAIL* and *KDM6B* are highly expressed in invasive breast carcinomas and are associated with tumor recurrence, metastasis, and decreased relapse-free survival^42^. Thus, we hypothesize that targeting H3K27me3 demethylases, which are potential therapeutic targets in other solid tumors like castration-resistant prostate cancer^43^, in combination with DNA hypomethylating agents may synergize to reduce recurrence.

### HH signaling and Polycomb complexes

HH signaling is another important developmental pathway deregulated by epigenetic mechanisms in breast tumorigenesis. Promoter hypomethylation of the HH ligand, *SHH* or its downstream receptor, *PTCH*, leads to increased ligand-dependent activation of the pathway and uncontrolled cell division driving cancer progression^37^ (Fig. 2). Moreover, HH signaling induces the expression of *PCGF4* (BMI1), a component of the PRC1 complex, to promote self-renewal of normal and tumorigenic mammary stem cells^44^. Breast cancer stem cells are linked to endocrine therapy resistance, however, it is still controversial whether the emergence of stem-like properties in resistant cells is due to expansion of pre-existing niche tumor cells or a dynamic reprogramming mediated by epigenetic changes. We believe that targeting epigenetic agents such as DNMTs to restore HH antagonistic regulation in combination with drugs that directly target HH signaling can potentially modulate cancer stem cell survival and differentiation. Such two-step strategies combining different classes of agents mediate a process known as directed phenotype switching were previously reported to sensitize resistant melanoma cells to lineage-specific therapy^45^. Recent data suggest that inhibitors against EZH2, the enzymatic core of the PRC2 complex, which is already in advanced clinical trials in multiple tumor types including triple-negative breast cancer, mediates de-repression of the GATA3-ERα signaling axis, inducing a luminal-like phenotype that is sensitive to endocrine therapy agents such as fulvestrant^46^. These results indicate that there is a therapeutic precedent to targeting Polycomb proteins, particularly PCGF4, to direct a phenotypic switch in endocrine-resistant breast cancer stem cells to an endocrine therapy sensitive state.

Altogether, these observations indicate that epigenetic mechanisms that play crucial roles in normal development are altered in neoplastic tissues and are, therefore, attractive candidates as biomarkers and therapeutic targets.

### Endocrine therapies target the oncogenic E2-ERα axis

Steroid hormone signaling was first correlated to breast cancer progression in 1896 when surgical removal of both ovaries of breast cancer patients resulted in tumor regression, providing rationale for endocrine therapy^47^. Endocrine therapy, the standard of care for ER^+^ breast cancer, refers to those interventions that suppress estrogen production as well as strategies that target ERα directly and comprises three main categories: selective estrogen receptor modulators (SERMs), selective estrogen receptor degraders (SERDs), and aromatase inhibitors (AIs). In addition, next-generation ERα targeting therapies are now in clinical trials as single agents or in combination with other drugs in ER^+^/HER2^−^ metastatic breast cancer^48^.

Tamoxifen was the first clinically approved ERα-targeted agent and has been the principal treatment option in both early and advanced breast cancer patients for over three decades. Tamoxifen is a SERM that competes with E2 for ERα binding and prevents coactivator recruitment mediated by the LBD of ERα. It can also promote activation of the AF1 domain through a ligand-independent mechanism, resulting in weak transcriptional activation in E2-deprived conditions and an incomplete block in E2-stimulated conditions in vitro^49^. These agonistic effects are associated with ERα activation via post-transcriptional modifications such as phosphorylation of serine 118 (pS118) in the AF1 domain by CDK7, MAPK, and mTOR^50^. Despite the success of tamoxifen therapy, one third of women treated with tamoxifen for 5 years will have recurrent disease within 15 years^51^. Nevertheless, because most of these patients retain ERα expression, they remain sensitive to SERDs like fulvestrant.

Fulvestrant disrupts ERα dimerization and nuclear localization, resulting in its degradation and a complete block of ERα-mediated transcriptional activity. Fulvestrant-mediated immobilization of ERα in the nuclear matrix is associated with the repression of transcription and subsequent degradation of ERα^52^. A phase III trial with luminal breast cancer patients who did not previously receive hormone therapy demonstrated that fulvestrant treatment results in superior progression-free survival compared with AIs^53^. Nevertheless, poor physicochemical properties and the need for muscular administration limit its clinical potential^52^. Currently, new orally available SERDs and a novel group of ERα-targeting agents that combine SERM and SERD features are under clinical development^54^ (Table 1).

**Table 1:** Targeting epigenetic mechanisms deregulated in endocrine-resistant breast cancer as new therapeutic avenues.

Mechanism of resistance	Deregulated epigenetic factor or process	Effect	Alternative therapeutic strategies	References
Imbalance in ERα co- regulators	*NF1* loss	Increased MEK-ERK signaling due to the loss of NF1 GTPase activity	MEK inhibitors (binimetinib) + fulvestrant	^77,117^
		Enhanced ERα transcriptional activity		
		E2 hypersensitivity		
		Increased cyclin D1 expression	CDK4/6 inhibitors	^56^
	*NCoR1* loss	Aberrant histone acetylation and deacetylation by HATs and HDACs	Selective inhibitors to HATs	^86^
Hormone-independent growth	*KMT2C* loss	Re-localization of ERα to AP-1-regulated genes	Fulvestrant	^92^
			CDK4/6 inhibitors	^56^
Constitutive activation of ERα	*ESR1* mutations	Ligand-independent reactivation of ERα via phosphorylation of S118	Fulvestrant	^66^
			CDK7 inhibitor (THZ1)	^66,67^
		Stabilization of ERα in an agonist conformation	Fulvestrant	^54^
			BET inhibitor (JQ1)	
		ERα recruitment to chromatin independent of E2	HDAC inhibitor (vorinostat)	
	*ESR1* fusion proteins	Hormone-independent growth	CDK4/6 inhibitors	^68^
		ERα cistrome reprogramming		
	Differential coactivator recruitment with mutant ERα (SRC-1/2/3 and KMT2C/D)	Enhanced proliferation and increased transcription of ERE-containing target genes	Pan-SRC small molecule inhibitor + oral SERD AZD9496	^65^
			KMT2C/D targeted inhibitors	
Downregulation of ERα	*ESR1* promoter methylation	Loss of ERα expression	HDAC inhibitors (entinostat, vorinostat)	^83,84^
			DNA hypomethylating agents (decitabine, 5- aza)	
Activation of mitogenic signaling pathways	KMT2D phosphorylation by AKT	Inhibition of KMT2D enzymatic activity	PI3K inhibitors (alpelisib)	^93^
		Reduced H3K4me1/2 at enhancers and impaired ERα and FOXA1 chromatin binding		
	EZH2 and DNMT1 phosphorylation by AKT	Switch between Polycomb-mediated gene repression and DNA methylation via inhibition of EZH2 enzymatic activity and DNMT1 stabilization	DNA hypomethylating agents (decitabine, 5-aza)	^94^
			PI3K inhibitors (alpelisib)	
		Global reduction of H3K27me3 and increased DNA methylation	HDAC inhibitors (entinostat, vorinostat)	
Alterations in TFs and chromatin remodeling complexes	*FOXA1* mutations	Redistribution of FOXA1 binding from active to de novo enhancers containing AP-1 to sustain hormone-independent growth and promote metastasis	Small molecule inhibitor against FOXA1 downstream target HIF-2α	^70,71,81^
	*ARID1A* mutations	Reduced ERα and FOXA1 binding to chromatin	EZH2 inhibitors (synthetic lethality)	^80^
			HDAC inhibitors (vorinostat)	
		Luminal-to-basal phenotype switch due to limited chromatin accessibility and binding of TFs that control luminal cell fate	BET inhibitors	
			PI3K/AKT inhibitors (buparlisib, GSK690693)	

In post-menopausal women, E2 is no longer synthesized in the ovaries. Instead, it is produced from the aromatization of testosterone and androstenedione in several tissues including the liver, subcutaneous fat, and the stroma surrounding normal breast cells, as well as by breast epithelial cells and fibroblasts of primary breast tumors. AIs act to reduce elevated E2 levels in breast cancer tissue through the inhibition of aromatase activity and can be classified as steroidal or non-steroidal. While steroidal AIs bind irreversibly with aromatase, non-steroidal AIs bind competitively and reversibly with aromatase. Two reversible non-steroidal AIs (letrozole, anastrozole) and one irreversible steroidal AI (exemestane) are currently approved for clinical use^55^. Compared to tamoxifen, which is typically prescribed for pre-menopausal breast cancer patients, fulvestrant and AIs are mainly reserved for post-menopausal cases alone or in combination with other endocrine or targeted agents such as CDK4/6 inhibitors.

**Fig. 3:**
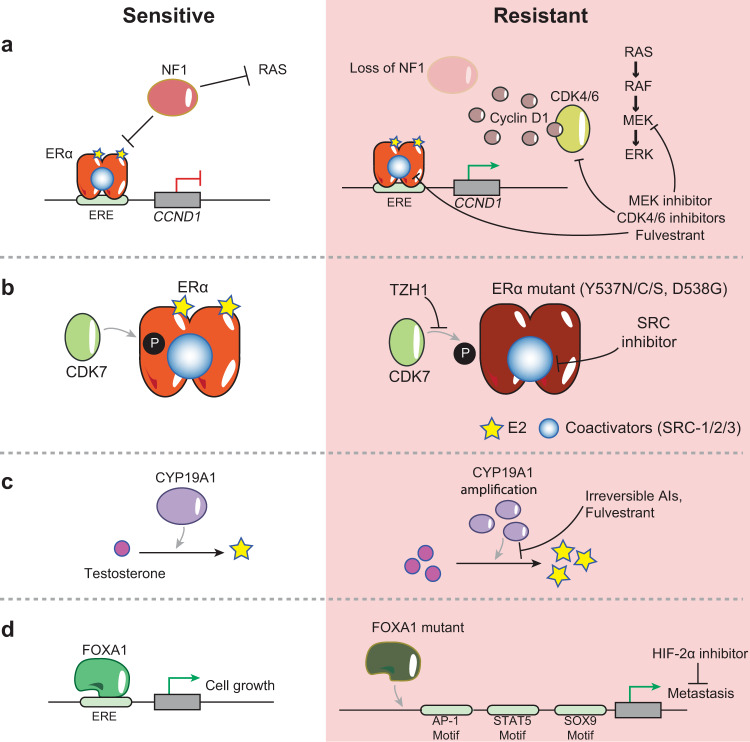
Illustrations of selected genomic alterations that mediate therapeutic resistance in ER^+^ breast cancer. **a** *NF1* is a GTPase that (1) inhibits RAS activation of the MAPK pathway and (2) functions as a corepressor of ERα at the *CCND1* gene, which encodes cyclin D1. Loss of *NF1* in treatment-resistant cells results in increased activation of the MAPK signaling pathway and overexpression of cyclin D1, which promotes G1/S transition by activating CDK4/6. MEK inhibitors and CDK4/6 inhibitors can overcome MAPK and CDK4/6 overexpression, respectively. Fulvestrant can also be used in cases where there is loss of *NF1*. **b** In treatment-sensitive ER^+^ breast cancer, ERα dimerizes upon binding to E2 and is phosphorylated by CDK7. *ESR1* mutations often occur within the LBD of resistant cells, leading to constitutively active ERα mutants that mediate gene expression through coactivator interactions independent of E2. Proposed therapeutic strategies include inhibition of CDK7 with THZ1 and inhibition of SRC coactivators. **c** *CYP19A1* codes for aromatase, the enzyme that converts testosterone into estrogen. *CYP19A1* overexpression is often acquired in patients that relapse after AI treatment, leading to increased estrogen and treatment resistance. Patients with *CYP19A1* overexpression can be treated with irreversible steroidal AIs instead of reversible AIs as well as with fulvestrant. **d** *FOXA1* is often amplified or mutated in treatment-resistant ER^+^ breast cancer, leading to increased FOXA1 activity and redistribution of FOXA1 to de novo enhancers. These de novo sites are enriched near TF motifs such as AP-1, STAT5, and SOX9, and promote a metastatic transcriptional program. Targeting FOXA1 directly is challenging. However, specific inhibitors can target FOXA1 downstream genes that mediate metastasis, such as HIF-2α.

While ERα is the primary oncogenic driver in ER^+^ breast cancer cancers, other genetic alterations such as cyclin D1 overexpression in 50% of breast cancers (Fig. 3a) and *CDKN2A* loss, contribute to disease progression and therapeutic response. For instance, cyclin D1 overexpression leads to increased activation of CDK4/6 as well as phosphorylation of RB, triggering cell-cycle progression through G1/S. After decades of endocrine monotherapy, the approval of targeted therapies against mTOR (everolimus), PI3K (alpelisib), and CDK4/6 (palbociclib, ribociclib, abemaciclib) led to significant progress in disease management. Multiple clinical trials demonstrated the efficacy of CDK4/6 inhibition^55,56^ and, as a result, CDK4/6 inhibitors alone or in combination with AIs (letrozole) or fulvestrant are established as standard-of-care options for both endocrine-sensitive and endocrine-resistant ER^+^/HER2^−^ metastatic breast cancers.

### Mechanisms of endocrine therapy resistance and potential alternative strategies

Despite the efficacy of endocrine therapy in the treatment of ER^+^ breast cancer, resistance arises in about 25% of the patients with early-stage disease and in almost all patients who develop metastasis, leading to poor clinical outcome^57^. Endocrine therapy resistance can be categorized as intrinsic (de novo) or acquired. Patients with advanced breast cancer typically exhibit progression at different sites that are clonally different and result from the selection of genetic alterations under therapeutic pressure^58^. This selective pressure leads to expansion of clones harboring mutations in the drug target itself, as well as in mitogenic signaling pathways and genes that encode for epigenetic factors. Moreover, microenvironmental conditions such as hypoxia may alter the epigenetic landscape and contribute to convergent evolution of the disease, especially since epigenetic enzymes are also nutrient and oxygen sensors. Specifically, clones with mutations in epigenetic machineries exhibit defects in transcription and DNA repair and replication, which lead to malignant self-renewal, differentiation blockade, and evasion of cell death, all promoting tissue invasiveness. Overcoming these outcomes is a major challenge in the ER^+^ breast cancer therapeutic arena. We discuss several mechanisms of resistance in detail in the following sections.

### Alterations of *ESR1* and genes involved in estrogen-mediated signaling

Endocrine therapy targets the tumor cell’s dependency on ERα for growth and survival. As a result, escape mechanisms to bypass drug inhibition center around accumulation of alterations in ERα and its downstream targets. In most patients, ligand-independent reactivation of ERα is the main mechanism of resistance^48^. Constitutive ERα activation can be mediated through gained mutations in *ESR1* (encoding ERα) and represents a leading driver of acquired resistance. Most ERα mutations are located at two adjacent amino acids in the LBD: tyrosine at position 537 mutated to either asparagine, cysteine, or serine (ERα^Y537N/C/S^) and aspartic acid at 538 mutated to glycine (ERα^D538G^). From a structural point of view, these mutations stabilize ERα in an agonist conformation leading to a constitutively active state^59^ (Fig. 3b). *ESR1* mutations are found in only about 1% of primary tumors but are detected in ~20–40% of metastases after endocrine therapy and correlate with poor response to AI and tamoxifen^57,58,60,61^. The near exclusive detection of *ESR1* mutations in metastatic breast cancer after AI therapy suggests a potential selection of rare, resistant clones under the pressure of endocrine treatment. However, the origin of these mutant clones is still under debate. It is not clear whether they arise from an undetectable pre-existing clone in treatment naïve primary tumors or whether they are acquired during treatment. A single-cell transcriptomics approach by Hong et al. identified small subsets of a treatment naïve population exhibiting a pre-adaptive phenotype^62^, suggesting that single-cell level techniques should be applied in patient diagnoses and disease monitoring to better assess response to therapy. Regardless of the origin, clones harboring *ESR1* mutations potentially have a selective advantage over endocrine-sensitive clones and expand to become predominant over the course of endocrine therapy^57^. As such, more precise and continuous monitoring of treatment responses is greatly warranted. Interestingly, *ESR1* mutations can be found in the circulating tumor DNA (ctDNA) of metastatic breast cancer patients that relapse after AI treatment^63^. ctDNA is a non-invasive source for monitoring response to therapy and is used to characterize the genetic features of tumors^64^. Such innovative techniques enable the detection of rare, sub-clonal mutations, such as those of *ESR1*^64^, emphasizing the importance of studying genetic alterations during disease evolution.

Numerous studies in the past years focused on discovering novel therapeutic strategies for the treatment of breast cancers harboring *ESR1* mutations. As a result of these efforts, it is now established that continuous ERα signaling promotes hormone-independent growth and is associated with a unique transcriptional network involved in growth factor signaling and metastasis^65^. ERα coregulators, activating kinases, and epigenetic modifying enzymes are essential for the growth of *ESR1* mutants^65–67^. Thus, they represent potential preclinical candidates for treating *ESR1* mutant-bearing tumors (Table 1).

*ESR1* gene fusion events represent another type of genetic alteration that is enriched in metastatic ER^+^ breast cancer and are considered to be new drivers of resistance. *ESR1* chromosomal translocation events result in proteins whereby the LBD of ERα is replaced by another protein. Notable examples include oncogenic TFs from the fusion of *ESR1* exons 1–6 with the C-terminal domains of *YAP1* or *PCDH11X*. These proteins are functionally active and, like *ESR1* mutations, induce the expression of ERE-containing target genes in a ligand-independent manner to sustain growth and metastatic progression. Since *ESR1* fusion proteins lack the LBD, tumors bearing these alterations are insensitive to endocrine therapies. Interestingly, targeting downstream ER signaling events with agents such as the CDK4/6 inhibitor palbociclib was demonstrated to suppress growth in vitro and in a PDX model of *ESR1*-exon6-*YAP1* fusion^68^. YAP1 binds to the *CDK6* promoter and contributes to CDK4/6 therapy resistance in patients with loss of the tumor suppressor *FAT1*. However, the N-terminal domain of YAP1, responsible for its biological effects, is not part of the *ESR1* fusion protein and its oncogenic contribution is not known.

Similar to *ESR1* activating mutations, genetic alterations in *CYP19A1*, the gene that encodes for aromatase, are acquired in patients that relapse after AI treatment, resulting in its increased enzymatic activity and E2-independent ERα binding to target genes (Fig. 3c). Aromatase overexpression leads to autonomous ERα activation and cellular invasion through an extensive epigenetic reprogramming^69^.

Gene amplification and missense mutations activate *FOXA1* and are reported in 6% and 10% primary and metastatic ER^+^ tumors, respectively^70^, resulting in genome-wide enhancer reprogramming in endocrine-resistant breast cancer cells. Interestingly, *FOXA1* mutations are mutually exclusive with *ESR1* mutations^58^. In tamoxifen resistance models, FOXA1 chromatin binding is redistributed from active enhancers to de novo enhancers containing AP-1 during acquisition of resistance and are enriched for TF binding motifs (e.g., STAT5 and SOX9) to promote metastasis^70^ (Fig. 3d). TFs such as FOXA1 are challenging therapeutic targets and are considered to be undruggable. Multiple approaches were explored to target various aspects of TF biology including expression levels, protein–protein interactions, and DNA-binding dynamics^71^, though no promising candidates emerged. In the meantime, downstream targets and effectors of TFs are proving to be potential alternatives. For instance, targeting the predominant FOXA1 downstream target, HIF-2α, and its premetastatic transcriptional program with small molecule inhibitors can circumvent endocrine resistance in patients with overexpressed FOXA1 or its associated signaling^70^ (Fig. 3d).

### Cell-cycle alterations in endocrine-resistant breast cancer

Changes in cell-cycle control are frequently linked to drug resistance. Moreover, *CCND1* amplification and high CDK4 levels in tumors correlate with endocrine resistance, though the loss of *RB* is rare^58^. Despite the efficacy of CDK4/6 inhibition, a subset of cancers (10–20%) remain insensitive and a large percentage (70–80%) becomes resistant after 12–36 months of therapy^72,73^. Resistance to CDK4/6 inhibition is characterized by loss of tumor suppressors such as *RB* and *FAT1*. Notably, loss-of-function mutations of *FAT1*, a Hippo pathway receptor, are observed in 2% and 6% of primary and metastatic tumors, respectively, and result in increased CDK6 expression due to the recruitment of YAP and TAZ to the *CDK6* promoter to drive G1/S progression^74^. In addition, hyperactivation of RTK-RAS signaling and aberrant activation of CCNE1-CDK2, a CDK4/6 downstream effector, restores RB phosphorylation and drives resistance as well as reduces response to palbociclib^73^. There are >100 active clinical trials testing efficacy of CDK4/6 inhibitors across many cancer types and treatment strategies, exemplifying its value as a candidate for future cancer therapy. More specifically, in breast cancer, administration of CDK4/6 inhibitors is actively being explored alone or in combination with endocrine therapy or immunotherapy agents (NCT03425838, NCT03285412, NCT03294694, NCT04318223).

The dependency of breast cancer cells on ERα signaling for cell survival and growth can be bypassed via mutually exclusive genetic alterations in mitogenic signaling pathways. For instance, *MAPK* mutations are associated with poor response to endocrine therapy and significant reduction in the duration of response to AIs and SERDs. Activation of the PI3K/AKT signaling pathway is also commonly observed in resistant tumor cells. Indeed, PI3K is the most frequently altered pathway in breast cancer and is essential for cell growth, proliferation, survival, and metabolism. Moreover, *AKT* activation and overexpression, as well as *PTEN* loss, is correlated with worse prognosis and tamoxifen resistance^75^. As a result of these observations, the PI3K inhibitor alpelisib was approved for advanced breast cancers alone or in combination with fulvestrant while others, such as buparlisib, are currently in trials (NCT01339442).

### Alterations of ERα coregulators

Genetic alterations can disrupt the balance between ERα coactivators and corepressors and are associated with poor prognosis and endocrine therapy resistance. Tamoxifen induces a conformational change of ERα that blocks coactivator recruitment and favors the recruitment of corepressors such as NCoR1 and NF1 (neurofibromin). *NCoR1* and *NF1* inactivating mutations or deletions are among the genetic alterations most frequently found in metastatic ER^+^ breast cancer^76^. For instance, *NF1* drives endocrine therapy resistance through the combined effects of loss of its GTPase activity and ERα transcriptional corepressor role, and its levels are associated with response to either endocrine therapy agents alone or combination with CDK4/6 or MEK inhibitors^77^ (Fig. 3a and Table 1).

### Epigenetic factors that contribute to endocrine-resistant breast cancer

**Fig. 4:**
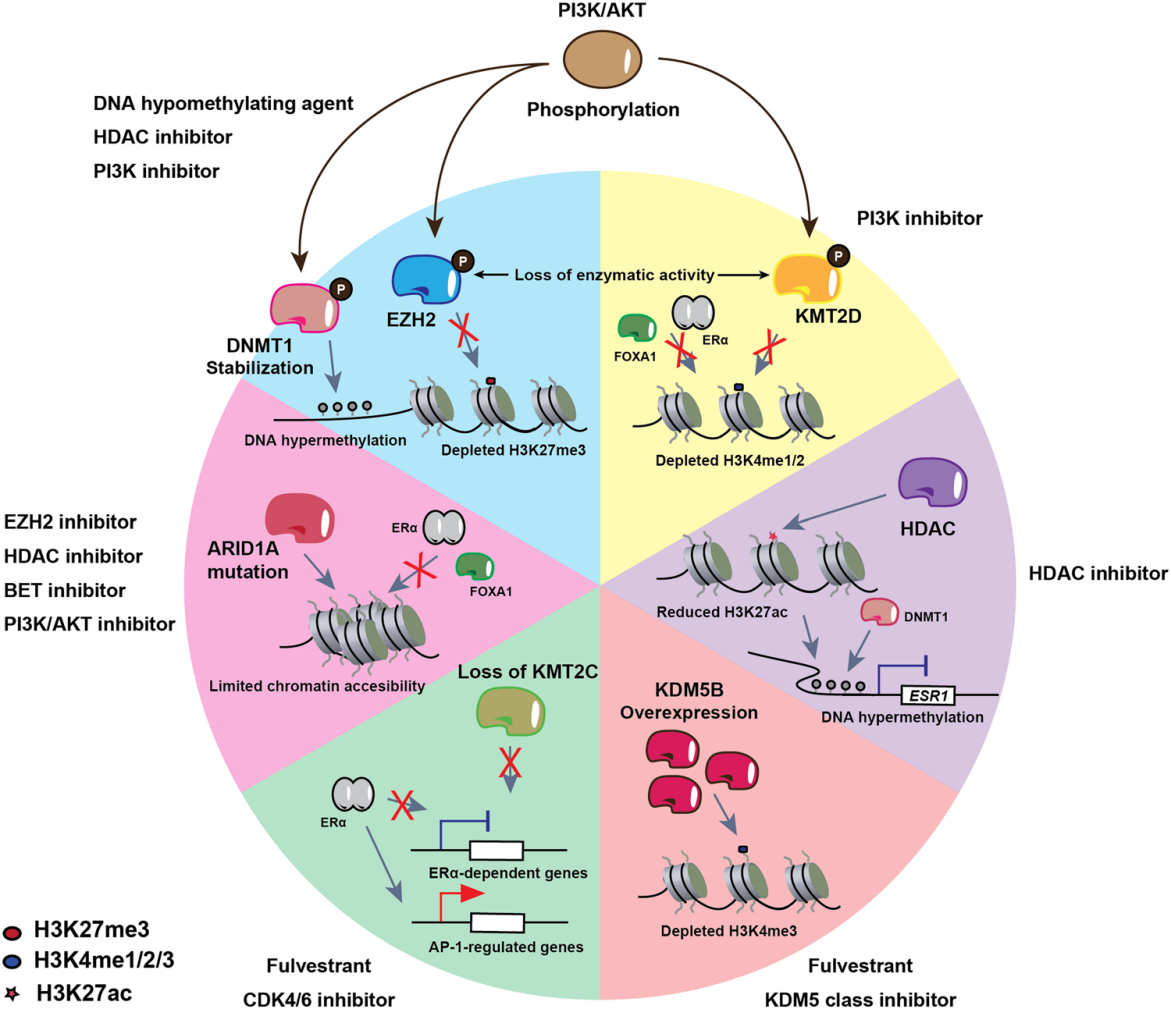
Schematic depiction of various dysregulated epigenetic pathways in treatment- resistant ER^+^ breast cancer that are potential targets for novel epigenetic therapeutics. Blue and yellow panels: the PI3K/AKT pathway can phosphorylate DNMT1, which stabilizes it on the chromatin, leading to maintenance of DNA hypermethylation. EZH2 is also phosphorylated by PI3K/AKT, which depletes H3K27me3 genome-wide. In addition, KMT2D phosphorylation by PI3K/AKT depletes H3K4me1/2, which decreases FOXA1 (and therefore, also ERα) chromatin binding, leading to hormone therapy resistance. These aberrant epigenetic pathways can be targeted by PI3K inhibitors. Furthermore, DNMT1 stabilization and EZH2 inhibition can be targeted by DNA hypomethylating agents and HDAC inhibitors, respectively. Purple panel: HDAC recruitment to the *ESR1* promoter leads to reduced H3K27ac, which results in DNMT1-mediated promoter hypermethylation and drug resistance via *ESR1* downregulation. HDAC inhibition (entinostat) can be used to reverse HDAC-mediated *ESR1* downregulation. Orange panel: KDM5 (KDM5A/B) is a family of histone H3 lysine 4 demethylases associated with therapeutic resistance in different cancer types. Increased activity of KDM5 enzymes leads to reduction in H3K4me3 levels and, as a result, increased transcriptional heterogeneity. Particularly, high KDM5B expression levels are associated with poor prognosis in ER^+^ breast cancer. Inhibitors to modulate the activity of KDM5 family members can improve the response to endocrine therapy agents such as fulvestrant. Green panel: loss of KMT2C redistributes ERα to AP-1-regulated genes to promote hormone-independent but ERα-dependent transcription, suggesting that treatment with SERDs (fulvestrant) and CDK4/6 inhibition may be viable therapeutic options. Red panel: *ARID1A* is a component of the SWI/SNF complex, and its mutation leads to limited chromatin accessibility for ERαα and FOXA1 at genes that regulate luminal cell fate, as well as promotes a switch from a luminal phenotype to a basal-like phenotype. *ARID1A* mutations can be targeted with EZH2, HDAC, BET, and PI3K/AKT inhibitors.

Whole-genome sequencing studies demonstrated that epigenetic factors are among the most commonly mutated genes in human cancers. Of these, inactivating mutations and loss of SWI/SNF subunits are the most frequent genetic alterations across many cancer types. In breast cancer, *ARID1A* determines breast luminal lineage fidelity and endocrine therapy sensitivity. Loss-of-function mutations in *ARID1A* are enriched in the endocrine-resistant metastatic setting, suggesting that they may also mediate endocrine resistance, with the latter reflected in the poor response to SERDs in patients with *ARID1A* mutations. Mechanistically, *ARID1A* loss reduces chromatin accessibility and binding of TFs that control luminal cell fate as well as reduces ERα and FOXA1 binding to chromatin (Fig. 4). Xu et al. hypothesized that prolonged ERα suppression may induce emergence of clones with *ARID1A* inactivating mutations to promote a luminal-to-basal phenotype switch^78^. Importantly, this switch is observed in the clinic where ER^+^ tumors treated with endocrine therapy undergo reprogramming to a basal-like phenotype, lose ERα expression, and become resistant to hormone therapy. The increased frequency of *ARID1A* mutations in endocrine-resistant breast cancer as well as its prevalence in other cancers amplifies the need for targeted therapeutic strategies against *ARID1A* mutant cancers (Table 1).

One of the therapeutic paradigms explored in *ARID1A* mutant cancers is synthetic lethality, which refers to the lethal effect of the simultaneous alteration of two genes which, when individually perturbed, do not impair cell viability^79^. For instance, in ovarian cancer, *ARID1A* mutations and EZH2 inhibition are synthetically lethal, an effect that is further potentiated by HDAC2 inhibition (Fig. 4). In *ARID1A* deficient cells, HDAC2 is recruited to ARID1A/EZH2 co-target genes such as *PIK3IP1*, an inhibitor of the PI3K/AKT signaling, leading to aberrant activation of this mitogenic pathway^80,81^. These two events, i.e., *ARID1A* loss-of-function and activated PI3K/AKT signaling, are commonly observed in endocrine-resistant breast cancer cells. Thus, we suggest that targeting EZH2 in *ARID1A*-mutated breast cancer could be a valid therapeutic option to explore (Fig. 4).

### Epigenetic avenues in the endocrine therapy road

Endocrine and targeted therapies were demonstrated to successfully eradicate the bulk of breast tumors but failed to target a small subset of the population that eventually drive relapse and therapeutic resistance. Compounding factors such as tumor genomic instability provide adaptability to a variety of stressors, including the selective pressure imposed by therapeutic agents, as discussed above. As a result, extensive patient stratification and customized lines of therapy will be necessary to mitigate the high mortality of patients with ER^+^ metastatic breast cancer.

Epigenetic dysregulation is a major contributing factor to tumorigenesis and drug resistance. To date, epidrugs are mainly confined to hematological malignancies with little success in solid tumors^82^. However, lack of efficacy in solid tumors can be attributed to the “one size fits all approach” used. The plasticity associated with epigenetic reprogramming increases the overall fitness of cancer cells, making customized cancer treatment markedly more complex. Several preclinical and clinical evidences support the synergistic effects of epidrugs with different therapeutic modalities including immunotherapy (Box 1), radiotherapy^82^, and endocrine therapy (discussed below). Indeed, development of small molecules that target chromatin regulators is one of the most active areas of current drug discovery efforts. Interestingly, recent efforts suggest that RNA modifications machineries are important in cancer progression and therapeutic resistance, demonstrating their potential as candidate targets for new therapies (Box 2).

Currently, DNA methylation and histone acetylation are the most explored epigenetic changes that occur during cancer progression and resistance. For example, in ER^+^ preclinical models, small molecule inhibitors targeting HDACis (entinostat and vorinostat) and DNA hypomethylating compounds (decitabine and 5-azacytidine) were explored as re-sensitizing agents to endocrine therapy^83,84^. In addition, multiple mechanisms of action were proposed for DNMT inhibitors (DNMTis) including demethylation of tumor suppressors and a novel viral mimicry mechanism (discussed in Box 1).

In addition, epigenetic dysregulation is a common occurrence in endocrine-resistant breast cancer. For instance, promoter hypermethylation of *ESR1* results in loss of ERα expression in about 20% of the patients that progress through tamoxifen^85^. Entinostat (HDACi) and letrozole (AI) can restore expression of ERα and aromatase in ER^−^ breast cancer cell lines, resulting in growth inhibition and demonstrating the efficacy of epigenetic intervention to induce endocrine therapy sensitivity^85^ (Fig. 4). However, restored ERα expression has never been observed in the clinical setting, suggesting that other repressive mechanisms are involved in *ESR1* silencing.

Tamoxifen-resistant cells are also characterized by increased acetylation of histones, TFs, and heat shock proteins catalyzed by HATs such as P300/CBP. Interestingly, in castration-resistant prostate cancer, a selective catalytic inhibitor of p300/CBP, A-485, inhibits the androgen receptor transcriptional program^86^. It remains to be tested whether targeting the balance between HAT and HDAC activities is similarly effective against ERα-mediated transcription in breast cancer. However, even selective class I HDACis (entinostat) modify the activity of several protein complexes containing HDACs, limiting the therapeutic benefit and resulting in off target effects and toxicity. Aiming to overcome these limitations, drugs inhibiting the context-specific enzymatic activity of HDACs are in development. Notably, Corin, a small compound that targets the enzymatic activity of both HDAC1 and LSD1 in the CoREST complex, is currently under study in solid tumors such as melanoma^87^ and glioma^88^.

Epigenetic agents also activate cell death mechanisms in response to endocrine therapy. Tamoxifen treatment induces autophagy of ER^+^ breast cancer cells, which promotes survival and contributes to the emergence of tamoxifen-resistant breast cancer. This phenotype can be combated with the combination of HDACi and tamoxifen, which predominantly redirects these cells into apoptosis by downregulating *BCL2* and inducing expression of the pro-apoptotic proteins BAX and BAK^89^. These evidences paved the way for several clinical trials with HDACis in combination with exemestane (NCT02820961, NCT00676663) and tamoxifen (NCT00365599, NCT01194427).

Moreover, epigenetic therapies such as HDACis show promising results in combination with tamoxifen to restore endocrine sensitivity^90^ and are currently in clinical trials in combination with CDK4/6 inhibitors (ribociclib, NCT04315233) and AIs (exemestane, NCT02820961).

New evidences demonstrate that mutations in epigenetic factors, such as histone methyltransferases, are common events and drive increased interest in the generation of other epidrugs targeting a wide array of chromatin regulators. For instance, the histone methyltransferase, *KMT2C*, is considered one of nine driver genes most commonly mutated in hormone receptor-positive metastatic breast cancer^76^. Loss of *KMT2C* results in downregulation of ERα-dependent gene expression and a re-localization of ERα to AP-1-regulated genes to sustain hormone-independent growth^91,92^ (Fig. 4). *KMT2C*-depleted cells retain ERα dependency and are sensitive to SERDs, suggesting that fulvestrant may be a therapeutic option for patients with *KMT2C* mutations (Fig. 4).

Signaling pathways such as PI3K/AKT can promote cancer cell survival through crosstalk with epigenetic factors. Aside from its oncogenic role, PI3K signaling regulates the breast cancer epigenome in which KMT2D is phosphorylated by AKT, thus inhibiting its methyltransferase activity. The resulting reduction of H3K4me1/2 at enhancers impairs ERα and FOXA1 chromatin binding, ultimately leading to endocrine therapy failure^93^ (Fig. 4). Similarly, in breast cancer cells, phosphorylation of EZH2 by AKT impairs its enzymatic activity, leading to depletion of H3K27me3. PI3K/AKT signaling simultaneously stabilizes DNMT1, which results in the maintenance of DNA hypermethylation^94^ (Fig. 4). These findings illustrate the redistribution of repressive epigenetic modifications in response to the same signaling pathway. Oncogenic signaling through PI3K/AKT has a direct effect on epigenetic balance, suggesting that the combination of PI3K/AKT inhibitors with epigenetic drugs is a candidate therapeutic strategy.

The recent development of powerful single-cell technologies allows us to address important questions such as the contribution of cell-to-cell variability to resistance. In ER^+^ breast cancer, KDM5B, a H3K4me3 demethylase, regulates cellular transcriptomic heterogeneity by decreasing the breadth of H3K4me3, which is a mark for high transcriptional fidelity and cell identity^95^. *KDM5B* is commonly amplified and overexpressed in luminal ER^+^ breast tumors resulting in increased transcriptomic heterogeneity that contributes to endocrine therapy resistance. Inhibitors of this enzyme were shown to increase sensitivity to fulvestrant in hormone-sensitive and endocrine-resistant cell lines^96^, suggesting their potential efficacy in the clinical setting.

Endocrine therapy reduces ER^+^ breast cancer mortality and recurrence but unfortunately, in many cases, the disease progresses to an incurable state. Genetic alterations enriched after endocrine therapy favor the use of combinatorial strategies with agents such as CDK4/6 or mTOR inhibitors which, too, results in resistance. Epidrugs provide the opportunity to rewire dormant cells to a proliferative and therapeutically sensitive state. Though there is much excitement surrounding use of these agents, there are still several obstacles in the road to their clinical use. Unfortunately, clinical results do not meet the expectations generated in the preclinical scenario in terms of efficacy and toxicity^97^. These observations could be attributed to epigenetic regulators having multiple substrates including histone and non-histone proteins. Scheduling and dosage of epidrugs should be carefully explored to enhance the benefits of combinatory therapeutic approaches and to reduce toxicity levels. The reversibility of epigenetic changes also remain a primary concern since re-expression of tumor suppressor genes can occur in the absence of the epidrugs or by redundant mechanisms.

Box 1 Epidrugs and immunotherapy Chromatin regulators coordinate the immune response. Epigenetic mechanisms are essential in the response to immunotherapy due to its roles in regulating expression of immune checkpoint inhibitors, infiltration of immune cells in the tumor, and changes in cytokine profile and antigen presentation^98^. In breast cancer, the utility of epigenetic modulation of the tumor microenvironment is still largely unexplored and a better understanding of the epigenetic processes that promote antitumor immunity is needed.

Epigenetic silencing of immune-associated genes is a determinant of an immune evasion signature. For instance, HDAC1 inhibition restores the sensitivity of prostate and breast cancer cells to the immune response coordinated by T cytotoxic lymphocytes^99^ and upregulates *PDL1* expression in melanoma and lung adenocarcinoma, thus potentiating the effects of anti-PD1-PDL1 therapy^100,101^. Interestingly, the SRC-3 inhibitor, SI-2, also elevates PDL1 expression^102^. In breast cancer patients that progress on non-steroidal AIs, the HDACi entinostat exhibits immunomodulatory action by reducing myeloid-derived suppressor cells (MDSCs) and increasing immunocompetent monocytes, resulting in improved overall survival^103^. Moreover, DNMTis restore expression of tumor suppressor genes and induce expression of endogenous retroviruses (ERVs) that lead to a viral mimicry state and potentiates the immune response^104^. Interestingly, histone methyltransferase inhibitors, HDACis, and agents such as vitamin C and CDK4/6 inhibitors are documented to modulate DNMTi activity and increase ERV expression. The resulting activation of ERVs as well as dsRNA sensing machineries lead to an inflammatory response driven by activated interferon signaling. Such direct regulation of the inflammatory response by epigenetic machineries expand the clinical use of epidrugs to restore and potentiate the response to immunotherapies.

While ER^+^ tumors are considered immunologically “cold” due to low tumor-infiltrating lymphocyte counts^55^, in endocrine-resistant tumors, immune checkpoint components such as IDO1 and LAG3 are upregulated and associated with poor prognosis, suggesting that strategies to engage the immune system response could improve patient outcome^105^. Moreover, estrogen signaling contributes to the immunosuppressive nature of breast cancer by driving the recruitment and functions of MDSCs^106^. All these evidences highlight the potential for combining epigenetic agents with immunotherapy and suggest an additional therapeutic benefit by adding endocrine therapy to these combinations. Several ongoing clinical trials are testing the effects of tamoxifen, vorinostat (HDACi), and pembrolizumab (anti-PD1) as an alternative to reverse endocrine therapy resistance through epigenetic rewiring of the immune response (NCT02395627, NCT04190056). Epidrugs, which target and prime the immune response, are shaping up as attractive strategies to improve existing therapies and overcome immunotherapy resistance.

Box 2 Epigenetic modifications of RNA in breast cancer Chemical modifications of protein coding and non-coding RNAs play important roles in various RNA biological processes, such as stabilization, decay, splicing, and nuclear export. Such modifications are reversible and a number of enzymes responsible for regulating them are reported^107^. Over the past decade, multiple studies provided evidence that dysregulation of RNA modifications is involved in the pathogenesis of cancer and resistance mechanisms.

The most abundant, evolutionarily conserved, and well-studied RNA modification is the methylation of adenosine at position 6 (N^6^-methyladenosine, m^6^A). m^6^A is found in many RNA species including mRNA, long non-coding RNA, miRNA, and rRNA^108^. m^6^A is essential for the maturation and function of these RNAs as well as their interaction with RNA binding proteins^109^. Deposition of m^6^A is carried out by a methyltransferase complex composed of METTL3, METTL14, and WTAP, and is removed by the RNA demethylases FTO and ALKBH5. Notably, ALKBH5 is an oncoprotein in breast cancer cells. Under hypoxic conditions, HIF-1α and HIF-2α induces ALKBH5 expression, which leads to enhanced mRNA stability of pluripotency factor genes such as *NANOG* and promotes the self-renewal and proliferation of breast cancer stem cells by increasing the stability of *NANOG* mRNA^110^.

Aside from m^6^A, methylation of cytidine residues at position 5 (5-methylcytosine, m^5^C) and adenosine residues at position 1 (N^1^-methyladenosine, m^1^A) are also implicated in cancer progression. For example, *YBX1*, which binds m^5^C, is highly expressed in breast cancer patients and is characterized as an oncogene^111^. Intriguingly, YBX1 not only interacts with RNA but also ERα in luminal breast cancer cells^112^, suggesting that RNA modifiers may regulate transcription through direct interaction with TFs in addition to indirect regulation through RNA modification. ALKBH3, a demethylase for m^1^A, promotes mRNA stability of CSF1, which regulates the density of tumor-associated macrophages and CD3^+^ T lymphocytes via its demethylation activity and leads to poor prognosis in breast cancer^113,114^. Furthermore, the 5’ cap structure of RNAs is also methylated. For instance, the methyltransferase MePCE can methylate the ncRNA *7SK* which interacts with the P-TEFb complex to confer invasion potential of breast cancer cells^115^.

tRNAs are also extensively modified. The methylation at the uridine 34 (U34) wobble position is involved in regulating base pairing and translation of mRNAs. The U34 modification to 5-methoxycarbonylmethyl-2-thiouridine is mediated by a protein complex consisting of ELP1, ELP3, CTU1, and CTU2. Interestingly, these factors are upregulated in non-invasive and invasive breast cancers and required for the efficient translation of DEK, which regulates specific pro-metastatic transcripts in mouse models of invasive breast cancer^116^.

The translation of these discoveries into the clinical setting is just beginning. Currently, several new strategies in cancer therapy combine epigenetic agents for DNA or histone protein modifiers with hormone therapies or chemotherapies^98^. Clinical studies with epigenetic agents targeting RNA modifiers should be further explored as they are emerging as significant contributors to cancer progression and resistance to current therapies.

### Concluding remarks and unresolved questions

Endocrine therapy has inarguably proven itself to be an indispensable option in the treatment of hormone-responsive breast cancers. However, there remains a dire need to develop approaches to attack the seemingly inevitable resistant phenotype. Recent developments in the epidrug arena are testimony to the burgeoning new era of epigenetic-based therapies to screen and treat multiple diseases, including breast cancer. As is the case in all aspects of research, new discoveries raise new questions and some of these key unresolved questions include:

1. What are the characteristics of the cell of origin in breast tumors?
2. Are epigenetic mechanisms mediating clonal selection at different metastatic sites?
3. What is the role of the mesenchymal niche in breast cancer progression? Which epigenetic mechanisms contribute to maintenance of the breast tumor microenvironment?
4. Can we exploit the dynamic nature of epigenetic changes to design short-term therapeutic strategies in efforts to avoid selection toward a resistant phenotype, or are the underlying mechanisms of epidrugs also contributing to the emergence of resistance?
5. Can we use epigenetic signatures to monitor disease progression and the response to therapy?
